# The Impact of Scientific and Technical Training on Improving Routine Collection of Antenatal Care Data for Maternal and Foetal Risk Assessment: A Case Study in the Province of South Kalimantan, Indonesia

**DOI:** 10.1155/2018/9240157

**Published:** 2018-09-13

**Authors:** Dewi Anggraini, Mali Abdollahian, Kaye Marion, Supri Nuryani, Fadly Ramadhan, Rezky Putri Rahayu, Irfan Rizki Rachman, Widya Wurianto

**Affiliations:** ^1^School of Science (Mathematical and Geospatial Sciences), College of Science, Engineering, and Health, RMIT University, GPO Box 2476, Melbourne, VIC 3001, Australia; ^2^Study Program of Mathematics, Faculty of Mathematics and Natural Sciences, University of Lambung Mangkurat (ULM), Ahmad Yani Street, Km. 36, Banjarbaru, South Kalimantan 70714, Indonesia; ^3^Study Program of Statistics, Faculty of Mathematics and Natural Sciences, University of Lambung Mangkurat (ULM), Ahmad Yani Street, Km. 36, Banjarbaru, South Kalimantan 70714, Indonesia; ^4^Ulin Public Hospital, 43 Ahmad Yani Street, Km 2.5, Banjarmasin, South Kalimantan 70233, Indonesia; ^5^Abdi Persada Midwifery Academy, 365 Sutoyo S. Street, Banjarmasin, South Kalimantan 70115, Indonesia

## Abstract

**Objectives:**

First, to assess the impact of scientific and technical training on midwives' abilities in collecting and recording the results of routine antenatal care examinations. Second, to explore midwives' views with regard to factors affecting their abilities to successfully complete the data documentation tasks.

**Methods:**

The study was conducted in South Kalimantan, Indonesia (April 2016-October 2017). Nineteen urban and rural midwives were selected. Access to antenatal care information on 4,946 women (retrospective cohort study) and 381 women (prospective cohort study) was granted. A descriptive and exploratory design was used to describe midwives' abilities and challenges pertaining to timely collection and recording of results concerning antenatal care examinations.

**Results:**

Scientific and technical training has significantly improved the average amount of recorded antenatal care data (from 17.5% to 62.1%, p-value < 0.0005). Lack of awareness, high workload, and insufficient skills and facilities are the main reasons for the database gaps.

**Conclusions:**

The training has equipped midwives with scientific knowledge and technical abilities to allow routine collection of antenatal care data. Provision and adequate use of this information during different stages of pregnancy is crucial as an evidence-based guideline to assess maternal and foetal risk factors to ending preventable mortality.

## 1. Introduction

Antenatal care (ANC) utilisation is highly recommended as a preventative action to improve pregnancy outcomes. Access to this service has been identified as one of the most effective interventions to prevent or manage complications and adverse birth outcomes [1–3]. ANC services provided across Indonesian healthcare centres are expected to comply with a quality integrated ANC standard to improve maternal and child health offerings, including recording and reporting the results of ANC examinations [4, 5]. This investment can provide sufficient information to be analysed and evidence to be used for informed planning, decision making, and monitoring policy progress to end preventable maternal and neonatal mortality [6–8].

Adequate use of ANC information and its systematic analysis during different stages of pregnancy is crucial to monitoring, detecting, and assessing the risks and preventable factors linked to maternal and neonatal mortality. In Indonesia, the access to timely, complete, and reliable data on pregnancy-related outcomes and the causes and the impacts of interventions remain challenging. This hinders planning programs, decision making, and allocating resources appropriately to reduce maternal, foetal, and neonatal mortality [5, 9–11]. Improvement of the ANC data availability, consistency, and quality during pregnancy can help medical practitioners detect the risks of abnormal delivery; consequently, proper interventions can be initiated in a timely manner [7, 8, 12–14].

In the Indonesian ANC model, midwives are the key practitioners across provinces (87.8% of medical practitioners) [15, 16]. They are expected to provide a comprehensive and integrated ANC service to pregnant women and document the results of examinations in local health recording and reporting systems, such as pregnancy registers, mothers' medical cards, and maternal and child health (MCH) booklets [5]. They are also expected to detect early signs of potential complications and abnormalities during pregnancy and delivery and provide appropriate interventions or referrals in a timely manner. Nevertheless, their abilities in documenting the results of ANC examinations have been reportedly low, with a 20% rate in hospitals and a 42.5% rate in primary healthcare (PHC) centres [11]. Unrecorded or unavailable local data on maternal, foetal, and neonatal care have been acknowledged as a major cause of hampering evidence-based interventions to track, review, and assess the causes and preventable factors associated with maternal and neonatal mortality [13, 17].

This study has assessed the impact of scientific and technical training on midwives' abilities in collecting and recording the results of routine ANC examinations in local pregnancy registers. Particularly, midwives were recruited who work in urban and rural PHC centres as these are the most locally recommended and cost-effective first level of healthcare in Indonesia. The study also explored midwives' views with regard to factors affecting their abilities to complete the data documentation tasks.

## 2. Methods

### 2.1. Research Design

A descriptive and exploratory design using both quantitative and qualitative methods was used. The study was carried out in two phases: quantitative design (phase 1) and qualitative design (phase 2). During the quantitative phase, a review of local pregnancy registers was conducted. The purpose of this phase was to assess and compare midwives' abilities in collecting and recording the results of recommended ANC examinations (Table 1) during service provision. This took place on two occasions: before hands-on scientific and technical training (retrospective cohort study using current manual pregnancy registers) and after the training program (prospective cohort study using electronic pregnancy registers). Meanwhile, during the qualitative phase, i.e., after the training program, electronic questionnaires were distributed to the midwives. This phase was used to gather information from the participating midwives regarding their views on challenges of documenting the results of ANC examinations in a timely manner.

### 2.2. Setting

The study was conducted in the province of South Kalimantan, Indonesia, between April 2016 and October 2017. The locality is one of the five provinces recording the highest neonatal mortality rate [11, 18, 19].

### 2.3. Participants

Nineteen midwives were recommended by the Provincial Health Department and Midwifery Association to participate in this study. They had been rendering antenatal and midwifery services for a minimum of five years at 19 PHC centres comprising 14 public health centres (PKMs) and 5 private midwifery clinics (BPMs). These centres are distributed throughout all administrative areas of the province (2 urban and 11 rural areas).

### 2.4. The Research Instrument and Data Collection

#### 2.4.1. Phase 1: Quantitative Design

*Hands-On Scientific and Technical Training. *Scientific and technical training was initiated and conducted amongst Indonesian urban and rural PHC's midwives (21-22 May 2016) to enhance the existing investment program in midwives. The program was intended to reduce maternal and neonatal mortality [9–11, 20]. The training aimed to educate and update midwives with scientific knowledge and technical abilities to allow for routine monitoring, measuring, collecting, and electronically recording of the significant maternal and foetal characteristics during ANC. This initiative was also meant to improve the availability, quantity, quality, and use of ANC information to strengthen routine maternal, foetal, and child health information systems and quality of care [3, 6, 11, 13, 17]. Additionally, the information can be utilised to monitor the progress of policies and programs in ending the preventable deaths due to prematurity, stillbirths, and low birth weight (LBW) [7, 17].

The training was divided into two sessions: scientific and technical. In the scientific domain, the maternal and foetal characteristics that play a vital role in evidence-based intervention decisions to reduce neonatal mortality were discussed. The session also covered scientific reasons for the significance of these characteristics and how their measurements are used to make evidence-based interventions to prevent neonatal mortality. This scientific introduction gave the participating midwives better insights into the importance of complete performance of such measurements and recording of results routinely from the start of pregnancy to delivery time.

An electronic pregnancy register was introduced in the technical part of the training. Each ANC category and its characteristics (Table 1) involved in the electronic register were technically and thoroughly explained and discussed to reach a consensus among the midwives. A demonstration of how to appropriately record and manage the data was also performed. This session provided the representative midwives better knowledge and skills of how to record the results of standard ANC measurements electronically and appropriately.

**Figure 1:**
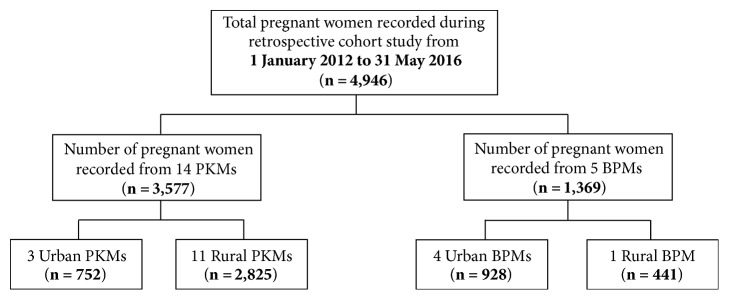
Description of retrospective data.

*Manual Pregnancy Register. *During the training, the participating midwives were asked to provide the current manual pregnancy registers (1 January 2012–31 May 2016) available at PHC centres where they were employed. The structure and content of these registers were then closely examined to match and track the recommended ANC characteristics suggested in Table 1. These records were then entered into the developed electronic pregnancy register for quantitative analyses by local graduates and research students who had experiences in the area of data entry. To improve the quality of the data processing task, the team in charge of data entry was trained to understand the content of the manual and electronic registers. This was followed by face-to-face and online communication between the principal investigator, the data collection team, and the midwives to minimise data entry error. The access to manually recorded ANC information on 4,946 women who enrolled, received care, and gave birth in the centres was granted (Figure 1).

*Electronic Pregnancy Register. *A bilingual electronic pregnancy register was created using a standard platform (Microsoft Excel) containing 12 categories of recommended ANC examinations listed in Table 1. This creation was based on current national application in conjunction with additional characteristics that have not been included but which are recommended nationally [4, 21–23] and internationally [7, 24–38].

The ANC categories are listed in the first column of Table 1. The objective of this tabulation is to stratify maternal and foetal measurements that are routinely undertaken during ANC service. The second column represents the recommended national and international characteristics under each ANC category. In the third column, unrecorded characteristics of the recommended ANC examinations are given. In the developed electronic register, new columns to record these characteristics were created. We proposed that these characteristics should be included in the current ANC data recording and reporting systems in Indonesia.

**Figure 2:**
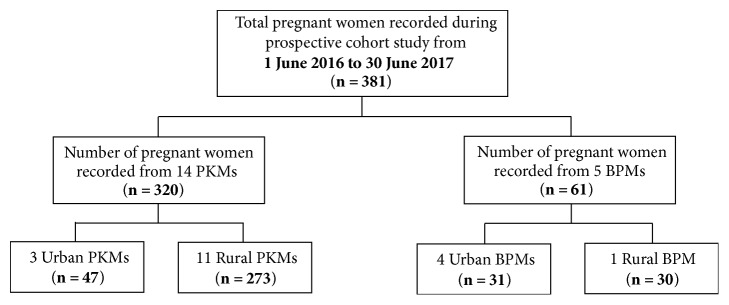
Description of prospective data.

At the end of the training sessions, the representative midwives agreed to participate in our prospective cohort study (1 June 2016–30 June 2017). By following the national standard operational procedures of ANC, the midwives were expected to longitudinally monitor and measure the recommended ANC examinations (Table 1) and record the results into the developed electronic pregnancy register in a timely manner. Online communication between the principal investigator and the midwives was conducted to improve the quality of the data processing task and minimise data entry error. Therefore, the access to electronically recorded ANC information on 381 women who enrolled, received care, and gave birth in the centres was granted (Figure 2).

#### 2.4.2. Phase 2: Qualitative Design

*Electronic Questionnaires. *After the training, an electronic feedback questionnaire was distributed to the participating midwives through email and social media platforms. The questionnaire covered questions on the current manual systems of recording and reporting ANC examination results, accessibility to the existing health information systems, and feedback on the training program. The questionnaire responses were used to assess the performance of the current ANC documentation systems, the potential challenges to complete the tasks, and the impact of the training program. The questionnaire responses were electronically collected between August and October 2016. A discussion forum was also conducted through a social media platform, both as personal communication among the midwives to clarify unclear specific responses and as a group to clarify common related issues of ANC data recording and reporting systems. In this study, 19 midwives' questionnaire responses were considered as preliminary data in the analysis, so as to enhance the future qualitative aspect of the study.

### 2.5. Statistical Analyses

Descriptive statistics were deployed to assess the performance of routine ANC data collection, particularly in documenting the key recommended maternal and foetal characteristics (Table 1). This was done by calculating the amount of available records of the identified ANC characteristics in both manual (retrospective study) and electronic (prospective study) pregnancy registers at each PHC centre. A two-sample t-test was used to compare the performance of data documentation process, before and after the training program, across PHC facilities. Data management and analyses were performed using Microsoft Excel 2010 and Minitab 17.

Analyses of the emerging codes, categories, and themes were conducted to investigate the causes of current ANC database gaps. The qualitative data were analysed using nonparametric statistics, frequencies, cross tabulations, and percentages. Cramer's V test was used to determine the degree of association between midwives' responses regarding factors affecting their ability to successfully complete the ANC data collection tasks. Data management and analyses were performed using Microsoft Excel 2010 and SPSS 23.

### 2.6. Ethics Approval and Consent to Participate

This study is a part of doctoral degree research and has obtained two ethics' clearances:

1. The Ethical Committees of Medical Research, Medical Faculty, University of Lambung Mangkurat (ULM), Banjarmasin, South Kalimantan (Indonesia), on March 10, 2016, with registration number: 018/KEPK-FK UNLAM/EC/III/2016.
2. The Science, Engineering, and Health College Human Ethics Advisory Network (CHEAN) of Royal Melbourne Institute of Technology (RMIT) University, Melbourne, Victoria (Australia), on March 16, 2016, with registration number: ASEHAPP 19-16/RM No: 19974.

Research permissions were also obtained from the Indonesian provincial and local governments. Information about the project and a consent form for recruitment to the study were given to the selected midwives and pregnant women (prospective study), who all agreed to participate.

## 3. Results

### 3.1. Midwives' Characteristics

Overall, the average age of the participating midwives was 41 years (29-56 years). The results revealed that 4 (21.1%) midwives were 46 years of age or older, 13 (68.4%) were 36-45 years old, and 2 (10.5%) were 25-35 years old. The average working experience of midwives in antenatal and midwifery services was 19 years. Their experience ranged from six to ten years (*n* = 4; 21.1%), eleven to twenty years (*n* = 9; 47.4%), twenty-one to thirty years (*n* = 5; 26.3%), and thirty-one or longer (*n* = 1; 5.3%).

### 3.2. The Impact of Scientific and Technical Training on Improving Routine Collection of ANC Data

Scientific and technical training has significantly improved the average amount of recorded ANC data suggested in Table 1 across PHC providers based on a two-sample t-test (from 17.5% to 62.1%, p-value <0.0005) (Table 2).

**Figure 3:**
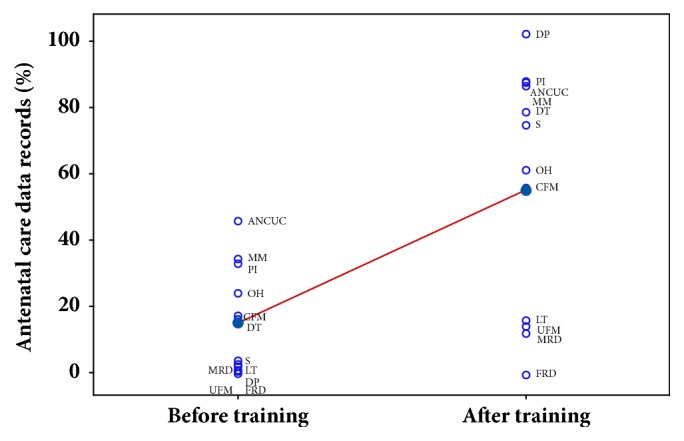
Individual plot of average ANC data records (%) before and after midwives' training across urban and rural PHC centres.

This significant improvement is presented in Figure 3 and listed in Table 3. The results show an overall improvement, particularly in documenting personal information, obstetric history, delivery plan, ANC utilisation criteria, maternal measurements, provision of supplements, clinical foetal measurements, and delivery time (from 0.8-47.9% to 57.4-99.2%). However, midwives' responsiveness on the importance of collecting and recording the results of laboratory tests, maternal risk detection, ultrasonic foetal measurements, and foetal risk detection suggest room for improvement (<12%).

### 3.3. The Improvement of Database Adequacy for Maternal and Foetal Risk Assessment

This analysis is accompanied by Tables 4-14 (Appendix A in the Supplementary material) that compare the abilities of midwives in recording the results of the recommended ANC examinations (Table 1) before and after the training program. The results can be used to assess the impact of training on improving database adequacy for maternal and foetal risk detection during pregnancy.

The ANC categories are presented in the first column together with their characteristics. The second and third main columns provide the type of PHC centres: PKMs and BPMs. These columns have been divided into two subcolumns to represent urban and rural areas. Urban areas (subcolumns 1 and 3) consist of 3 midwives representing PKMs and 4 midwives representing BPMs, respectively. Rural areas (subcolumns 2 and 4) comprising of 11 midwives representing PKMs and 1 midwife representing BPM, respectively. In each subcolumn, the amount of recorded ANC data (%) of the identified characteristics has been calculated. The relevant summary of detailed data collection's performance is given below.

#### 3.3.1. Maternal Risk Assessment

Maternal risks can be assessed from their personal information, obstetric history, delivery plans, maternal measurements, laboratory tests, and nutritional interventions/supplements.

*Personal Information (PI). *A significant improvement of midwives' abilities in documenting personal information is indicated in Table 4, particularly in data access to educational background, occupation, ownership of health insurance, ownership of MCH booklets, prepregnancy weight, prepregnancy height, prepregnancy BMI, and blood type (from 0.0-54.3% to 63.8-100.0%).

*Obstetric History (OH). *Midwives' competencies were improved in data collection tasks on obstetric history, particularly in recording the number of still births and premature births (Table 5). They were also more responsive in documenting gravidity, parity, number of deliveries, abortions, and live births, while a rural BPM's midwife collected substantially more data on prepregnancy contraception, pregnancy interval, the last birth attendance, the last TT immunisation, and the last mode of delivery (from 0.0% to 70-96.7%). However, midwives were less aware of the importance of documenting complication history (<17%) and chronic diseases and allergies (<7%).

*Delivery Plans (DP). *The performance of midwives in documenting information on birth preparedness was significantly improved across urban and rural centres (from 0.0-4.1% to 95.2-100.0%) (Table 6). This involved plans for birth attendance, birth place, birth companion, transportation, and blood donor.

*Maternal Measurements (MM). *The responsiveness of urban and rural midwives in collecting and recording the results of maternal measurements during pregnancy was significantly improved (Table 8). The examinations included anamnesis, patellar reflex, maternal MUAC, nutritional status, blood pressure, body temperature, pulse, and breath (from 0.0-88.0% to 66.1-100.0%). Improvement was also seen in the assessment of abdominal palpation and fundal height (from 0.0-69.8% to 42.5-100.0%).

*Laboratory Tests (LT). *The average amount of recorded laboratory data before (1.5%) and after (11.3%) the training program remained low (Table 3). However, urban and rural midwives have attempted to improve the data accessibility of laboratory test results. This is particularly true for the haemoglobin test (from 0.0-20.2% to 4.8-35.6%, Table 9) and the blood test (from 0.0-29.7% to 93.41-100.0%, Table 4), which are highly recommended to be routinely performed during pregnancy [5].

*Supplements (S). *Midwives' abilities in documenting the provision of supplements was substantially improved, particularly among rural midwives (from 0.0-42.5% to 81.0-100.0%) (Table 10), except TT immunisation records (< 45%). Less well documented was coverage and documentation of this characteristic across PHC facilities.

*Maternal Risk Detection (MRD). *Midwives have made efforts to improve information on maternal complications, yet the amount of records remained low (<26%) across PHC centres (Table 11). This resulted in a lack of documentation on appropriate interventions (<32%) and referral tasks (<15%).

#### 3.3.2. Foetal Risk Assessment

Foetal risks can be assessed through available information on foetal measurements based on clinical and ultrasonic methods.

*Foetal Measurements: Clinical (CFM) and Ultrasonic (UFM) Methods. *Overall, midwives' competencies have improved in clinically documenting foetal growth (Table 12). Rural midwives tended to provide more information on the number of gestation, foetal weight estimation, foetal heart rate, foetal presentation, and foetal station/descent level than urban midwives (from 0.0-61.0% to 57.3-100.0%). A rural BPM's midwife was more responsive in collecting and recording the results of foetal measurements through ultrasound (from 0.0% to 0.9 -70.4%).

*Foetal Risk Detection (FRD). *Comparable to maternal risk detection, the competence of midwives in gathering information on foetal complications was persistently low (< 1%) (Table 13). As a result, data documentation on appropriate interventions and referral tasks was also low (< 2%).

#### 3.3.3. ANC Service Utilisation and Pregnancy Outcomes

ANC service utilisation and pregnancy outcomes can be assessed through available ANC utilisation criteria and maternal and neonatal information recorded at delivery time.

*Antenatal Care Utilisation Criteria (ANCUC). *The responsiveness of urban and rural midwives in collecting and recording the criteria of ANC utilisation during pregnancy was significantly improved (from 47.9% to 91.0%) (Table 3). The criteria included measurement of GA, method of ANC enrolment, date of consultation, date of the next consultation, and number of ANC visits.

*Delivery time (DT). *The performance of midwives in documenting maternal and neonatal information at delivery time was significantly improved across urban and rural centres (from 14.0% to 75.8%) (Table 3). The information included GA (from 8.8-34.9% to 100.0%), newborn gender (from 17.3-67.1% to 95.7-100.0%), birth weight (from 18.0-68.5% to 100.0%), and survival status of mother and newborn (from 7.7-47.7% to 100.0%) (Table 14). However, data documentation on neonatal anthropometric characteristics and delivery complications should be further improved.

### 3.4. Midwives' Perspectives on Challenges in Timely Collecting and Recording the Results of ANC Examinations

Table 15 (Appendix B in the Supplementary material) is a cross tabulation analysis of midwives' opinions of the existing ANC data recording and reporting systems. The table includes their feedback on the scientific and technical training. The first column lists the main open questions with the respective categories of answers. The second column describes the percentage of responses across urban and rural PKMs and BPMs. The third column represents the percentage of responses for each category of identified answers. The last column shows the Cramer's V test value carried out to identify whether there is an association between midwives' responses with respect to factors affecting their ability to successfully complete the ANC data documentation tasks. A 5% significant level is used for the Cramer's V test.

Combination between pregnancy registers, mothers' medical cards, and MCH booklets are the most commonly used manual formats among midwives to collect and record ANC data (26.3-31.6%) (Table 15). Twenty-one per cent of the midwives believed that the design and infrastructure of these formats should be further improved. Supervision and monitoring on the completeness of ANC data have been routinely undertaken (78.9%). Almost half of the midwives have been trained for ANC data management. However, surprisingly, fewer than 20% of them were aware of and using the existing electronic database formats, either SIKDA Generic (5.3%) or PWS KIA Kartini (15.8%).

Poor recording and reporting systems (31.3%), unawareness of the pregnancy (25.0%), and time limitations (25.0%) were the main contributing factors for midwives not completing ANC data records (Table 15). Almost 60.0% of the midwives had to manually prepare multiple reports every month. Lack of awareness (46.2%), high workload (30.8%), and insufficient skills and facilities (15.4%) were the main reasons for the delay in collecting and reporting routine ANC data. Seventeen midwives (89.5%) responded to the effectiveness of the training and 52.6% of them responded to the importance of the developed electronic register, particularly if it could be computationally and automatically linked with the monthly ANC reporting formats.

## 4. Discussion

### 4.1. Age and Experience of the Midwives

Most midwives' ages ranged between 29 and 56 years, implying that the participants were senior midwives. The midwives' working experience ranged between 6 and 36 years in antenatal and midwifery services across urban and rural PHC facilities.

### 4.2. The Impact of Scientific and Technical Training on Improving Routine Collection of ANC Examination Results

Scientific and technical training has significantly equipped Indonesian urban and rural midwives, as pivotal health practitioners to ANC service [14, 42, 43], with the knowledge of the importance of ANC data documentation. It is documented that investments on continuous training among midwives are vital to ending preventable deaths [14]. The improvement of their basic midwifery care in documenting the results of ANC examinations was overall higher (62.1%) (Table 3) than the current national report (42.5%) [11]. This was particularly the case in collecting and recording the key quality characteristics of mother and foetus used to assess the risks during pregnancy.

Routine collection of ANC data is vital to improving maternal and foetal health. If good quality of care can be associated with its effective data collection during labour [14], then it is also crucial to having real-time data collection at different stages of pregnancy to optimize the quality of ANC. Such data collection is important to promoting evidence-based approaches to ANC for positive, transparent, and respectful pregnancy experiences and improvement of pregnancy outcomes [3].

Currently, local health registers rather than periodic household surveys are used to identify risk factors to reduce maternal and neonatal mortality [9, 10, 44]. With the adoption of a decentralisation policy in Indonesia [9, 10], access to routine and consistent local data collection of maternal and foetal measurements during ANC is urgently required. Given appropriate training, supervision, quality control, and technology to strengthen the records maintenance, local maternal, foetal, and neonatal health data can be used as reliable baseline information to improve the quality of care services [44].

### 4.3. The Improvement of Database Adequacy for Maternal and Foetal Risk Assessment

Access to individual (disaggregate) information on maternal and foetal health during pregnancy and at delivery is vital to strengthen accountability of routine health information systems. Our training has significantly improved midwives' responsiveness in consistently documenting the key characteristics of individual mother and foetus at different stages of pregnancy and delivery. The use of this information enables midwives to improve the quality of risk assessment tasks and pregnancy outcomes and to target informed planning, interventions, and referrals. It is for the purpose of preventing maternal, neonatal, and child mortality [8, 11, 14] and promoting equality [13], particularly in rural areas.

It is well documented that maternal lifestyle and chronic disease history are factors that contribute to spontaneous preterm births, stillbirths, and LBW [7, 14, 45] influencing maternal, foetal, and neonatal health [5]. Although tobacco and substance uses are recommended to be investigated during maternal assessment at every ANC visit [38], there are no specific columns, in the current manual pregnancy register, that allow midwives to document such information, except under the chronic diseases and allergies column. Therefore, we recommend additional columns be provided for recording smoking habits and alcohol consumption separately to improve maternal risk detection.

Midwives' unawareness in documenting information on a delivery plan potentially caused birth preparedness to be one of the remaining and emerging challenges in reducing maternal and neonatal mortality in Indonesia [11]. The training has significantly improved midwives' responsiveness in recording this information (from 0.8% to 99.2%) (Table 3). This would increase community demand for the use of care and consequently improve the quality continuum and effectiveness of obstetric care.

In the current national ANC standard, MUAC is measured once only, in the first trimester of pregnancy, to screen the risk of chronic energy deficiency or malnutrition among pregnant women [5]. We highly recommend that MUAC be measured routinely at different trimesters of pregnancy based on the following reasons. MUAC has a significant relationship with BMI which corresponds to maternal weight and height [46]; hence, it may be affected by the changes of maternal weight. MUAC is well documented to be a factor that contributes to spontaneous preterm births [7] and relates to LBW which is one of the main causes of neonatal deaths [5, 11]. This becomes a focus to end preventable stillbirths [8]. This recommendation can potentially be considered a new baseline for evaluation in the future, particularly in the effort of reducing neonatal mortality and achieving the target of Sustainable Development Goals (SDGs) 2030 [11].

Data collection of laboratory test results during pregnancy is crucial. Keeping these records is highly recommended [5, 7, 44] since it can be used as an information base to improve detection of infections caused by microorganisms during pregnancy, particularly for those who are living in vulnerable and epidemic areas [37]. For instance, maternal infections and anaemia are well-documented to be one of the risk factors in the occurrence of spontaneous preterm births [7], stillbirths [14], and neonatal deaths [11]. Although the training program has improved laboratory data accessibility, the average amount of records across PHC services remained low. These findings may indicate that some of the laboratory tests are not necessarily performed. For example, malaria, syphilis, and HIV tests should be routinely undertaken only in high-prevalence settings [5, 37, 47] where in our study population this was not the case. In addition, urine protein, blood sugar level, and tuberculosis tests are only performed when symptoms occur [5].

Midwives' competencies in documenting the provision of nutritional interventions/supplements (iron, folic acid, calcium, and vitamin C) for pregnant women were significantly improved after the training program (from 0.0-42.5% to 34.0-100.0%) (Table 10). Daily intake of these supplements is highly recommended to prevent anaemia, sepsis, LBW, preterm births, stillbirths, and preeclampsia; consequently, it improves pregnancy outcomes [3, 5, 14, 38, 48]. Attention should be directed to the documentation of TT immunisation across PHC centres which remained below 45%. This is due to the fact that the vaccination is not necessary if a pregnant woman has previously been vaccinated or if her TT immunisation is known [5, 38].

Serial ultrasonic scanning has been recommended in the national and international ANC standards as a means to detect foetal growth abnormalities [4, 24, 25, 38, 41, 49]. However, the fields to record such measurements do not exist in the manual pregnancy register (Table 12). These findings indicate that the ultrasonic method is not accessible in the current practice of ANC services across Indonesian PHC centres because it requires intensive resources [38]. Even if it was accessible, there were no columns to facilitate the documentation of the measurements. In the developed electronic register, we have provided such columns to enable a rural midwife who was trained for ultrasound to record the results of the examination during pregnancy even though the percentage of recorded data in the current ANC needs further improvement.

Information on maternal and foetal complications, which can be derived from risk assessment analysis during pregnancy, is vital to improving the quality of ANC service and positive pregnancy outcomes [3]. Midwives, as the pivotal practitioners to ANC, are expected to record this information based on their integrated analyses of maternal and foetal characteristics [5, 14]. Nevertheless, the amount of recorded information across PHC centres was not sufficient (0.0%) even after the training (6.5%) (Table 3). These results clearly indicate gaps in undertaking the risk analysis. The gaps are potentially due to the fact that midwives are required to record the signs of abnormalities only if they are present. The other significant reason is that screening tools, such as foetal growth charts, are currently not available to assist midwives in carrying out the systematic risk analysis during pregnancy.

### 4.4. Midwives' Perspectives on Challenges in Successfully Completing Routine ANC Data Documentation Tasks

Most midwives have reportedly complied with the standard ANC examination procedures in Indonesia [5]. However, some of them agreed that the design and infrastructure of the existing data recording and reporting formats need to be further improved to support accuracy and complete documentation, storage ability, and record maintenance. This result is similar to findings reported by other researchers [1].

The midwives stated that supervision and monitoring on ANC data collection have been initiated but were carried out irregularly. They were also trained for ANC data management yet many of them remained unfamiliar with the existing electronic applications, such as SIKDA Generic and PWS KIA Kartini. Lack of knowledge on these database systems was also described by other researchers [50].

The midwives informed several factors triggering incomplete and delayed ANC data recording and reporting tasks. These included high workload, lack of time for routine ANC examinations, limited skills and training, and lack of awareness about the importance of recording the examination results. This is consistent with those of Burke et al. [44] and Sibiya et al. [1] who found similar factors hindering complete and timely documentation of the recommended ANC examination results.

The midwives positively responded to the effectiveness of the training and the importance of keeping the results of ANC examinations electronically. They also recommended that the training should be routinely conducted among other midwives so that they have an equal chance to update their knowledge and improve their capabilities in timely recording and reporting ANC data from local to provincial and national levels. Ongoing education (raising awareness) and training might then be an integral part of investment programs in midwives to further reduce maternal and neonatal mortality [11, 20].

## 5. Conclusion

Risk assessment of maternal and foetal complications during pregnancy is vital to preventing potential adverse pregnancy outcomes. Adequate use of ANC information and its systematic analysis during different stages of pregnancy is crucial to assessing the prevalence of maternal and foetal risk factors. The statistical analysis shows that scientific and technical training has increased Indonesian midwives' awareness of the importance of monitoring and measuring the key characteristics of mother and foetus during pregnancy and at delivery as well as collecting and maintaining the records electronically.

Strong commitment and consistent education/training coupled with routine supervision of data documentation and records maintenance can significantly improve midwives' competencies to report the results of ANC examinations in a timely manner. This will lead to improvement of the quality and quantity of routine collection of ANC data, promote a reliable and transparent local data recording and reporting system as baseline information, and allow the task of vital data transformation. Consequently, the national health information system needs to be strengthened and made more reliable to be used as an evidence-based guideline in targeting appropriate resource planning and allocations, interventions, and referrals. The ultimate aim is to end preventable maternal and neonatal mortality.

## Figures and Tables

**Table 1:** List of recommended contents of ANC examinations.

**ANC category**	**Recommended/current ANC characteristics**	**Proposed ANC characteristics**
Personal information (PI)	Name, name of partner/husband, date of birth, address, contact number, educational background, occupation, religion, maternal age, date of the first registration/visit, ownership of health insurance, ownership of Maternal and Child Health (MCH) booklet, prepregnancy weight, prepregnancy height, and blood type.	Ethnicity/country of birth and prepregnancy body mass index (BMI).

Obstetric history (OH)	Gravidity, parity, number of deliveries, number of abortions, number of live births, obstetric complication history, chronic diseases and allergies, the last delivery date, the last menstrual period, and the estimated delivery date.	Number of stillbirths, number of premature births, prepregnancy contraception, distance between previous and current pregnancies, the last birth attendance, the last tetanus toxoid (TT) immunisation, and the last mode of delivery.

Delivery plans (DP)	Birth attendance, birth place, birth companion, transportation, and blood donor.	-

Antenatal care utilisation criteria (ANCUC)	Gestational age (GA), the method of antenatal care (ANC) enrolment, date of consultation, and date of the next consultation.	Number of antenatal care (ANC) visits.

Maternal measurements (MM)	Anamnesis, patellar reflex, weight, middle upper arm circumference (MUAC), nutritional status, blood pressure, and fundal height (FH).	Height, body mass index (BMI), body temperature, *blood pressure (column separation between systole and diastole records)*, pulse, breathe, and abdominal palpation (Leopold I, II, III, and IV).

Laboratory tests (LT)	Haemoglobin level, urine protein, syphilis, maternal urine reduction, blood sugar level, thalassemia, hepatitis B surface antigen, prevention of mother to child transmission (human immunodeficiency virus (HIV) test), rapid test (malaria), and tuberculosis.	*Haemoglobin level: before and after having iron tablets*, sputum acid resistant bacteria, and ankylostoma test.

Supplements (S)	Iron tablets and tetanus toxoid (TT) immunisation.	Folic acid, calcium, aspirin, and vitamin C.

Maternal risk detection (MRD)	Maternal complication, referral, and risk detector.	Intervention action.

Foetal measurements: clinical method (CFM)	Number of gestation, foetal weight estimation, foetal heart rate, foetal presentation, and foetal station/descent level (FS).	-

Foetal measurements: ultrasonic method (UFM)	Not available	Gestational age (GA) based on ultrasound scanning, crown-rump length, head circumference, abdominal circumference, biparietal diameter, femur length, humerus length, placenta localisation, foetal presentation, amniotic fluid index, foetal heart rate, and foetal weight estimation.

Foetal risk detection (FRD)	Not available	Foetal complication, intervention action, referral, and risk detector.

Delivery time (DT)	Gestational age (GA) at delivery time, last menstrual period age at delivery time, active phase I and II (date and time), active phase III management, breast feeding initiation, neonatal delivery (date and time), placenta delivery (date and time), new born gender, new born presentation, birth weight, birth length, head circumference, birth place and address, delivery complication, referral, birth attendance, integration programs, bleeding status, mode of delivery, and survival status (mother and new born).	Abdominal circumference, chest circumference, femur length, humerus length, and intervention action towards delivery complications.

Source: [4, 21–28, 30–33, 38–41].

**Table 2:** Two-sample t-test on the performance of ANC data collection before and after midwives' training across urban and rural PHC centres.

Treatment	Mean	Standard deviation	SE Mean	Estimate for difference	95% Confidence interval for difference		T value	P value	Pooled standard deviation
					Lower bound	Upper bound			
After training	62.1	40.4	1.7	44.6^*∗∗∗*^	40.5	48.8	21.1	<0.0005	35.0
Before training	17.5	28.5	1.2						

*∗∗∗*Significant at p-value < 0.0005.

*∗∗*Significant at p-value < 0.05.

*∗* Significant at p-value < 0.1.

**Table 3:** Average data records (%) of ANC category before and after midwives' training across urban and rural PHC centres.

**12 recommended ANC categories**	**Before training (**%**)**	**After training (**%**)**
Personal information (PI)	33.1	91.7
Obstetric history (OH)	25.9	64.4
Delivery plans (DP)	0.8	99.2
Antenatal care utilisation criteria (ANCUC)	47.9	91.0
Maternal measurements (MM)	32.3	82.1
Laboratory tests (LT)	1.5	11.3
Supplements (S)	5.0	69.9
Maternal risk detection (MRD)	2.4	6.5
Clinical foetal measurements (CFM)	18.0	57.4
Ultrasonic foetal measurements (UFM)	0.0	11.3
Foetal risk detection (FRD)	0.0	0.4
Delivery time (DT)	14.0	75.8

## Data Availability

The data used to support the findings of this study are included within the article.

## Abbreviations

ANC: Antenatal care
PHC: Primary healthcare
PKM: Public primary healthcare centre
BPM: Private primary healthcare centre (midwifery clinics)
LBW: Low birth weight
PI: Personal information
OH: Obstetric history
DP: Delivery plans
ANCUC: Antenatal care utilisation criteria
MM: Maternal measurements
LT: Laboratory tests
S: Supplements
MRD: Maternal risk detection
CFM: Clinical foetal measurement method
UFM: Ultrasonic foetal measurement method
FRD: Foetal risk detection
DT: Delivery time
GA: Gestational age
MCH: Maternal and child health
TT: Tetanus toxoid
MUAC: Middle upper arm circumference
BMI: Body mass index
HIV: Human immunodeficiency virus
FH: Fundal height
FS: Foetal station/descent level
SIKDA: Regional health information system
PWS KIA: Local monitoring system of maternal and child health
SDGs: Sustainable Development Goals.

## Supplementary Materials

Supplementary Materials Appendix A: performance of data collection on 12 categories of recommended ANC examinations across urban and rural PHC centres before and after midwives' training. Table 4: personal information. Table 5: obstetric history. Table 6: delivery plans. Table 7: antenatal care utilisation criteria. Table 8: maternal measurements. Table 9: laboratory tests. Table 10: supplements. Table 11: maternal risk detection. Table 12: foetal measurements: clinical and ultrasonic. Table 13: foetal risk detection. Table 14: delivery time. Appendix B: perceptions on challenges of timely collecting and recording the results of ANC examinations. Table 15: midwives' perceptions on the current ANC data recording and reporting systems and the training program.
