# Supplementary material for: The Impact of Scientific and Technical Training on Improving Routine Collection of Antenatal Care Data for Maternal and Foetal Risk Assessment: A Case Study in the Province of South Kalimantan, Indonesia
Source: J Pregnancy. 2018 Sep 13;2018:9240157. doi: 10.1155/2018/9240157 (PMC6158931; doi:10.1155/2018/9240157)
Supplement: Supplementary Materials — Appendix A: performance of data collection on 12 categories of recommended ANC examinations across urban and rural PHC centres before and after midwives' training. Table 4: personal information. Table 5: obstetric history. Table 6: delivery plans. Table 7: antenatal care utilisation criteria. Table 8: maternal measurements. Table 9: laboratory tests. Table 10: supplements. Table 11: maternal risk detection. Table 12: foetal measurements: clinical and ultrasonic. Table 13: foetal risk detection. Table 14: delivery time. Appendix B: perceptions on challenges of timely collecting and recording the results of ANC examinations. Table 15: midwives' perceptions on the current ANC data recording and reporting systems and the training program. [file 9240157.f1.pdf]

Appendix A Performance of data collection on 12 categories of recommended ANC examinations across urban and rural PHC centres before and after midwives' training

Table 4 Personal information

ANC category	PKMs				BPMs			
	Urban areas		Rural areas		Urban areas		Rural areas	
	%		%		%		%	
	Before training	After training	Before training	After training	Before training	After training	Before training	After training
**Total Pregnancy**	**752**	**47**	**2825**	**273**	**928**	**31**	**441**	**30**
**Personal information (PI)**								
Name	99.7	100.0	99.8	100.0	100.0	100.0	100.0	100.0
Name of partner/husband	99.5	100.0	93.2	100.0	99.1	100.0	97.5	100.0
Date of birth	0.0	93.6	1.2	84.3	0.1	54.8	0.0	100.0
Address	99.2	100.0	69.2	100.0	93.3	96.8	99.5	100.0
Contact number	0.7	27.7	0.0	41.4	7.1	48.4	83.7	96.7
Educational background	0.0	95.7	1.4	100.0	13.9	96.8	0.0	100.0
Occupation	0.0	100.0	0.7	99.6	32.3	100.0	0.0	100.0
Religion	0.0	100.0	2.3	99.6	29.4	100.0	0.0	100.0
Maternal age	99.3	100.0	72.7	100.0	95.0	100.0	99.1	100.0
Date of the first registration/visit	6.0	100.0	93.7	100.0	57.3	100.0	0.0	100.0
Ownership of health insurance	0.0	63.8	16.7	98.9	3.2	93.6	0.0	100.0
Ownership of Maternal and Child Health (MCH) booklet	53.7	100.0	54.1	100.0	8.4	96.8	0.0	100.0
Prepregnancy weight (kg)	44.9	97.9	11.7	100.0	5.7	100.0	0.0	100.0
Prepregnancy height (cm)	35.0	97.9	9.9	100.0	10.8	100.0	0.0	100.0
Blood type	29.7	97.9	18.2	93.4	4.1	93.6	0.0	100.0
Ethnicity/country of birth[#]	0.0	0.0	0.0	33.0	0.0	32.3	0.0	100.0
Prepregnancy Body Mass Index (BMI)[#] (kg/m^2)	54.3	97.9	35.5	100.0	10.9	100.0	0.0	100.0

[#]Currently not available in the current manual ANC register

Table 5 Obstetric history

ANC category	PKMs				BPMs			
	Urban areas		Rural areas		Urban areas		Rural areas	
	%		%		%		%	
	Before training	After training	Before training	After training	Before training	After training	Before training	After training
**Total Pregnancy**	**752**	**47**	**2825**	**273**	**928**	**31**	**441**	**30**
**Obstetric history (OH)**								
Gravidity	98.1	100.0	60.8	100.0	50.4	100.0	71.2	100.0
Parity	96.4	100.0	66.9	100.0	86.7	100.0	71.4	100.0
Number of deliveries	86.2	100.0	53.8	100.0	83.6	100.0	71.4	100.0
Number of abortions	86.6	100.0	40.0	100.0	78.9	100.0	71.4	100.0
Number of live births	77.8	100.0	46.1	100.0	35.6	100.0	71.2	100.0
Number of stillbirths#	0.0	100.0	0.0	100.0	0.0	100.0	0.0	100.0
Number of premature births#	0.0	100.0	0.0	100.0	0.0	100.0	0.0	100.0
Obstetric complication history	0.0	14.9	0.0	7.0	0.3	0.0	0.0	16.7
Chronic diseases and allergies	0.0	0.00	0.0	0.7	0.0	0.0	0.0	6.7
Prepregnancy contraception#	0.0	0.00	0.0	27.8	0.0	25.8	0.0	90.0
Distance between previous and current pregnancies#	0.0	0.0	0.0	22.0	0.0	25.8	0.0	73.3
The last birth attendance#	0.0	0.0	0.0	21.6	0.0	22.6	0.0	70.0
The last tetanus toxoid (TT) immunisation#	0.0	0.0	0.0	30.4	0.0	9.7	0.0	96.7
The last mode of delivery#	0.0	0.0	0.0	21.6	0.0	22.6	0.0	70.0
The last delivery date	0.0	72.3	1.1	73.6	1.5	32.3	0.0	70.0
The last menstrual period	36.2	100.0	31.3	100.0	73.4	100.0	29.3	100.0
The estimated delivery date	43.6	100.0	22.7	100.0	62.5	100.0	52.8	100.0

[#]Currently not available in the current manual ANC register

Table 6 Delivery plans

ANC category	PKMs				BPMs			
	Urban areas		Rural areas		Urban areas		Rural areas	
	%		%		%		%	
	**Before training**	**After training**	**Before training**	**After training**	**Before training**	**After training**	**Before training**	**After training**
**Total Pregnancy**	**752**	**47**	**2825**	**273**	**928**	**31**	**441**	**30**
**Delivery plan [DP]**								
Birth attendance	0.1	100.0	3.4	99.6	4.1	100.0	0.0	100.0
Birth place	0.3	100.0	0.0	100.0	3.8	100.0	0.0	100.0
Birth companion	0.0	100.0	0.0	100.0	3.9	100.0	0.0	100.0
Transportation	0.0	97.9	0.0	96.3	0.0	100.0	0.0	100.0
Blood donor	0.0	95.7	0.0	95.2	0.9	100.0	0.0	100.0

Table 7 Antenatal care utilisation criteria

ANC category	PKMs				BPMs			
	Urban areas		Rural areas		Urban areas		Rural areas	
	%		%		%		%	
	**Before training**	**After training**	**Before training**	**After training**	**Before training**	**After training**	**Before training**	**After training**
**Total Pregnancy**	**752**	**47**	**2825**	**273**	**928**	**31**	**441**	**30**
**Antenatal care utilisation criteria [ANCUC]**								
Gestational age (weeks)	96.5	99.5	92.1	98.1	77.4	70.1	59.6	100.0
Method of ANC enrolment	0.0	95.7	1.9	97.9	0.0	63.0	0.0	100.0
Date of consultation	53.3	97.9	91.9	98.1	75.3	73.2	69.2	100.0
Date of the next consultation	0.0	69.7	1.9	88.3	0.8	68.5	0.2	100.0
Number of ANC visits[#]	96.4	100.0	95.1	100.0	76.5	100.0	69.2	100.0

[#]Currently not available in the current manual ANC register

Table 8 Maternal measurements

ANC category	PKMs				BPMs			
	Urban areas		Rural areas		Urban areas		Rural areas	
	%		%		%		%	
	**Before training**	**After training**	**Before training**	**After training**	**Before training**	**After training**	**Before training**	**After training**
**Total Pregnancy**	**752**	**47**	**2825**	**273**	**928**	**31**	**441**	**30**
**Maternal measurements (MM)**								
Anamnesis	0.0	97.9	11.9	85.3	48.6	66.1	0.0	100.0
Patellar reflex	0.0	72.3	16.4	86.7	0.5	70.9	0.0	100.0
Weight (kg)	90.2	97.9	72.2	98.1	76.5	71.7	69.6	100.0
Height (cm)	79.4	97.9	56.7	98.1	55.5	71.7	5.2	100.0
BMI (kg/m^2)	78.5	97.9	70.8	98.1	53.3	71.7	5.2	98.6
Middle upper arm circumference (MUAC) (cm)	77.8	97.9	57.9	83.9	63.8	72.4	3.2	100.0
Nutritional status	77.7	97.9	59.2	83.9	63.8	73.2	3.2	100.0
Blood pressure (systole)[#] (mmHg)	88.0	97.3	68.8	97.8	76.5	70.9	69.6	100.0
Blood pressure (diastole)[#] (mmHg)	88.0	97.3	67.9	97.8	76.5	70.9	69.6	100.0
Body temperature[#](^{o}C)	0.0	72.9	0.0	97.8	8.2	70.1	0.0	100.0
Pulse[#]	0.0	76.1	0.0	97.8	14.7	70.1	0.0	100.0
Breath[#]	0.0	76.1	0.0	97.7	14.7	70.1	0.0	100.0
Abdominal palpation (Leopold I)[#]	0.0	96.8	0.9	89.1	26.6	71.7	0.0	100.0
Abdominal palpation (Leopold II)[#]	0.0	58.5	1.3	66.9	26.4	45.7	44.7	99.1
Abdominal palpation (Leopold III)[#]	0.0	56.9	0.6	69.2	19.4	44.9	34.9	66.7
Abdominal palpation (Leopold IV)[#]	0.0	55.3	0.5	66.9	14.2	43.3	36.7	67.1
Fundal height (cm)	0.0	62.2	16.5	65.8	62.0	42.5	69.8	63.5

[#]Currently not available in the current manual ANC register

Table 9 Laboratory tests

ANC category	PKMs				BPMs			
	Urban areas		Rural areas		Urban areas		Rural areas	
	%		%		%		%	
	Before training	After training	Before training	After training	Before training	After training	Before training	After training
**Total Pregnancy**	**752**	**47**	**2825**	**273**	**928**	**31**	**441**	**30**
**Laboratory tests (LT)**								
Haemoglobin level before having iron tables#	20.2	35.6	11.2	18.3	8.4	24.4	0.9	13.2
Haemoglobin level after having iron tables#	0.0	4.8	0.0	18.5	0.0	9.5	0.0	13.2
Urine protein	0.0	49.5	3.3	33.5	7.0	53.5	4.5	0.9
Sputum acid resistant bacteria#	0.0	3.2	0.0	0.7	0.0	0.8	0.0	0.0
Syphilis	0.0	2.7	0.7	0.3	0.0	0.8	0.0	0.0
Maternal urine reduction	19.0	47.3	0.1	23.6	0.2	45.7	0.0	1.8
Blood sugar level	0.0	26.1	1.1	7.1	0.1	8.7	0.0	0.5
Thalassemia	0.0	10.6	0.8	0.2	0.0	0.0	0.0	0.0
Hepatitis B surface antigen	0.0	23.4	0.0	2.6	0.0	7.1	0.0	0.9
Prevention of mother to child transmission (human immunodeficiency virus (HIV) test)	0.0	42.0	0.0	5.4	0.0	3.9	0.0	0.9
Rapid test (malaria)	0.0	21.8	0.0	18.6	0.0	0.0	0.0	0.5
Tuberculosis	0.0	2.7	0.0	2.9	0.0	0.0	0.0	0.0
Ankylostoma#	0.0	0.0	0.0	0.0	0.0	0.0	0.0	0.0

#Currently not available in the current manual ANC register

Table 10 Supplements

ANC category	PKMs				BPMs			
	Urban areas		Rural areas		Urban areas		Rural areas	
	%		%		%		%	
	Before training	After training	Before training	After training	Before training	After training	Before training	After training
**Total Pregnancy**	**752**	**47**	**2825**	**273**	**928**	**31**	**441**	**30**
**Supplements (S)**								
Iron tablets	0.0	88.8	42.5	90.8	1.2	65.4	0.0	100.0
Folic acid#	0.0	91.0	0.0	85.1	1.3	68.5	0.0	100.0
Calcium#	0.0	64.4	0.0	81.0	0.0	68.5	0.0	100.0
Aspirin#	0.0	18.6	0.0	82.8	0.0	65.4	0.0	100.0
Vitamin C#	2.5	34.0	0.9	86.7	1.6	67.7	0.0	100.0
Tetanus toxoid (TT) immunisation	14.8	28.2	36.6	30.3	18.3	44.1	0.0	17.4

#Currently not available in the current manual ANC register

Table 11 Maternal risk detection

ANC category	PKMs				BPMs			
	Urban areas		Rural areas		Urban areas		Rural areas	
	%		%		%		%	
	Before training	After training	Before training	After training	Before training	After training	Before training	After training
**Total Pregnancy**	**752**	**47**	**2825**	**273**	**928**	**31**	**441**	**30**
**Maternal risk detection (MRD)**								
Maternal complication	0.0	25.5	0.9	20.0	0.0	5.5	3.6	0.9
Maternal intervention action#	0.0	14. 9	2.4	10.5	31.1	4.7	0.0	0.0
Referral	0.1	0.0	0.0	14.7	0.5	0.8	0.5	0.9
Risk detector	0.0	0.0	0.0	1.5	0.0	2.4	0.0	1.4

#Currently not available in the current manual ANC register

Table 12 Foetal measurements: clinical and ultrasonic

ANC category	PKMs							BPMs
	Urban areas		Rural areas		Urban areas		Rural areas	
	%		%		%		%	
	Before training	After training	Before training	After training	Before training	After training	Before training	After training
**Total Pregnancy**	**752**	**47**	**2825**	**273**	**928**	**31**	**441**	**30**
**Foetal measurements: clinical method (CFM)**								
Number of gestation	0.0	56.4	25.5	78.5	0.0	62.3	0.0	100.0
Foetal weight estimation (g)	0.0	33.5	4.4	57.3	0.3	26.8	50.6	63.9
Foetal heart rate	0.0	50.0	20.9	65.6	28.6	41.7	61.0	77.2
Foetal presentation	0.0	43.6	24.4	61.1	12.2	40.2	54.4	70.3
Foetal station/descent level	0.0	50.0	24.2	58.7	0.1	40.2	54.2	70.8
**Foetal measurements: ultrasonic method (UFM)**								
Gestational age (GA) based on ultrasound scanning# (weeks)	0.0	0.0	0.0	3.7	0.0	1.6	0.0	70.4
Crown-rump length# (mm)	0.0	0.0	0.0	0.3	0.0	0.0	0.0	18.3
Head circumference# (mm)	0.0	0.0	0.0	0.1	0.0	0.0	0.0	55.3
Abdominal circumference# (mm)	0.0	0.0	0.0	0.4	0.0	0.0	0.0	57.1
Biparietal diameter# (mm)	0.0	0.0	0.0	0.0	0.0	0.0	0.0	54.8
Femur length# (mm)	0.0	0.0	0.0	0.2	0.0	0.0	0.0	49.8
Humerus length# (mm)	0.0	0.0	0.0	0.0	0.0	0.0	0.0	6.0
Placenta localisation#	0.0	0.0	0.0	1.1	0.0	1.6	0.0	58.9
Foetal presentation#	0.0	0.0	0.0	2.8	0.0	2.4	0.0	54.8
Amniotic fluid index#	0.0	0.0	0.0	0.7	0.0	1.6	0.0	0.9
Foetal heart rate#	0.0	0.0	0.0	1.2	0.0	2.4	0.0	50.7
Foetal weight estimation# (g)	0.0	0.0	0.0	2.6	0.0	2.4	0.0	39.3

#Currently not available in the current manual ANC register

Table 13 Foetal risk detection

ANC category	PKMs							BPMs
	Urban areas		Rural areas		Urban areas		Rural areas	
	%		%		%		%	
	Before training	After training	Before training	After training	Before training	After training	Before training	After training
**Total Pregnancy**	**752**	**47**	**2825**	**273**	**928**	**31**	**441**	**30**
**Foetal risk detection (FRD)**								
Foetal complication#	0.0	0.0	0.0	0.0	0.0	0.8	0.0	0.0
Foetal intervention action#	0.0	0.0	0.0	0.0	0.0	0.8	0.0	0.0
Referral#	0.0	0.0	0.0	0.1	0.0	1.6	0.0	0.0
Risk detector#	0.0	0.0	0.0	0.2	0.0	3.2	0.0	0.0

#Currently not available in the current manual ANC register

Table 14 Delivery time

ANC category	PKMs							BPMs
	Urban areas		Rural areas		Urban areas		Rural areas	
	%		%		%		%	
	Before training	After training	Before training	After training	Before training	After training	Before training	After training
**Total Pregnancy**	**752**	**47**	**2825**	**273**	**928**	**31**	**441**	**30**
**Delivery time (DT)**								
Gestational age (GA) at delivery time (weeks)	12.2	100.0	34.9	100.0	8.8	100.0	34.2	100.0
Last menstrual period age at delivery time (weeks)	0.1	97.9	32.4	99.6	13.6	96.8	0.0	100.0
Active phase I (date)	0.0	100.0	16.1	96.0	3.1	77.4	0.2	83.3
Active phase I (time)	0.0	70.2	15.5	93.8	2.9	71.0	0.0	76.7
Active phase II (date)	0.0	97.9	23.2	94.5	3.1	67.7	0.0	100.0

Active phase II (time)	0.0	70.2	15.3	93.4	3.0	67.7	0.0	80.0
Active phase III management	0.0	78.7	15.7	96.0	3.1	67.7	0.0	100.0
Breast feeding initiation	0.0	97.9	14.9	96.3	3.1	71.0	0.0	100.0
Neonatal delivery (date)	30.7	100.0	36.1	100.0	19.1	90.3	69.6	100.0
Neonatal delivery (time)	27.4	95.7	40.8	96.0	19.0	87.1	66.9	100.0
Placenta delivery (time)	0.0	93.6	34.9	95.6	10.9	67.7	69.6	100.0
New born gender	29.8	95.7	29.1	99.6	17.3	96.8	67.1	100.0
New born presentation	0.1	98.0	42.6	99.6	23.6	93.6	0.0	100.0
Birth weight (g)	35.2	100.0	31.3	100.0	18.0	100.0	68.5	100.0
Birth length (cm)	29.4	95.7	34.6	99.6	35.8	83.9	64.2	100.0
Head circumference (cm)	0.0	95.7	16.9	92.3	26.9	77.4	0.0	80.0
Abdominal circumference (cm)[#]	0.0	61.7	0.4	82.8	9.7	35.5	0.0	53.3
Chest circumference (cm)[#]	0.0	95.7	7.5	83.5	1.4	71.0	0.0	53.3
Femur length (cm)[#]	0.0	29.8	1.0	32.2	0.1	3.2	0.0	53.3
Humerus length (cm)[#]	0.0	29.8	0.5	43.2	0.4	3.2	0.0	53.3
Birth place	25.4	97.9	23.9	100.0	5.3	93.6	7.7	100.0
Address of birth place	23.4	95.7	6.7	96.7	4.4	90.3	0.0	100.0
Delivery complications	0.4	2.1	2.3	4.8	0.6	0.0	0.0	3.3
Intervention action	0.3	4.3	0.1	5.9	0.0	6.5	0.0	3.3
Referral	6.8	4.3	6.8	4.8	1.2	6.5	8.6	0.0
Birth attendance	12.9	95.7	30.5	97.8	11.4	93.6	69.6	100.0
Integration programs	0.1	25.5	10.5	71.8	3.1	38.7	0.0	100.0
Bleeding status (cc)	0.0	53.2	0.0	23.8	2.6	22.6	0.0	3.3
Mode of delivery	1.2	95.7	18.9	98.9	11.0	100.0	0.0	100.0
Survival status (mother)	25.7	100.0	29.9	100.0	11.2	100.0	7.7	100.0
Survival status (new born)	25.9	100.0	47.7	100.0	11.3	100.0	7.7	100.0

Appendix B Perceptions on challenges of timely collecting and recording the results of ANC examinations

Table 15 Midwives' perceptions on the current ANC data recording and reporting systems and the training program

Type of questions and category of answers	Responses (N = 19)				Total (%)	Cramer's V test value (p-value)
	Urban PKMs (% of Total)	Rural PKMs (% of Total)	Urban BPMs (% of Total)	Rural BPM (% of Total)		
Existing formats of recording ANC data (Q.1.1)						
Pregnancy registers	0.0	15.8	0.0	0.0	15.8	.523 (.211)
Pregnancy registers and MCH booklets	0.0	10.5	0.0	5.3	15.8	
Pregnancy registers and mothers' medical cards	0.0	5.3	5.3	0.0	10.5	
MCH booklets and mothers' medical cards	5.3	10.5	15.8	0.0	31.6	
Pregnancy registers, MCH booklets, and mothers' medical cards	10.5	15.8	0.0	0.0	26.3	
What need to be improved in the existing formats (Q2.2)						
Structure/layout/infrastructure	5.3	15.8	0.0	0.0	21.1	.515 (.240)
Time to complete	0.0	5.3	0.0	0.0	5.3	
All good	0.0	0.0	5.3	5.3	10.5	
No comment	10.5	15.8	10.5	0.0	36.8	
No response	0.0	21.1	5.3	0.0	26.3	
Existing methods of recording ANC data (Q1.3)						
Manual	0.0	36.8	15.8	0.0	52.6	.552 (.128)
Manual and computational	15.8	21.1	5.3	5.3	47.4	
Current procedure of reporting ANC data (Q1.4)						
BPM reports to PKM	5.3	0.0	10.5	5.3	21.1	.510 (.107)
PKM reports to health department	5.3	21.1	0.0	0.0	26.3	
BPM/village midwife reports to PKM, then PKM reports to health department	5.3	36.8	10.5	0.0	52.6	
Number of submitted reports (Q1.5)						
One	0.0	25.0	0.0	0.0	25.0	.505 (.525)
Two	0.0	16.7	0.0	0.0	16.7	
More than three	8.3	16.7	25.0	8.3	58.3	
Factors hindering the process of recording complete ANC data (Q1.6)						
Time limitation	6.3	0.0	12.5	6.3	25.0	.572 (.220)
Network limitation	0.0	6.3	0.0	0.0	6.3	
Poor recording and reporting systems	12.5	18.8	0.0	0.0	31.3	
Unawareness of pregnant women	0.0	25.0	0.0	0.0	25.0	
Combination	0.0	12.5	0.0	0.0	12.5	
Factors hindering the process of timely report ANC data (Q1.7)						
Lack of skills and facilities/tools	7.7	7.7	0.0	0.0	15.4	.543 (.261)
High workload	7.7	7.7	7.7	7.7	30.8	
Unawareness	7.7	38.5	0.0	0.0	46.2	
All good	0.0	0.0	7.7	0.0	7.7	
Supervision and monitoring to review the completeness of ANC data (Q1.8)						
Yes	15.8	47.4	15.8	0.0	78.9	.494 (.310)
No	0.0	10.5	5.3	5.3	21.1	
The implementation of SIKDA Generic (Q2.1.1)						
Yes	0.0	0.0	5.3	0.0	5.3	.463 (.206)
No	10.5	36.8	15.8	5.3	68.4	
No idea	5.3	0.0	0.0	0.0	5.3	
No response	0.0	21.1	0.0	0.0	21.1	
The implementation of PWS KIA Kartini (Q2.1.2)						
Yes	5.3	5.3	5.3	0.0	15.8	.273 (.898)
No	10.5	42.1	15.8	5.3	73.7	
No response	0.0	10.5	0.0	0.0	10.5	
Training experience on ANC data management/recording and reporting systems (Q2.3)						
Yes	10.5	31.6	5.3	0.0	47.4	.308 (.730)
No	5.3	21.1	15.8	5.3	47.4	
No response	0.0	5.3	0.0	0.0	5.3	
Response to the initiated scientific and technical training (Q3.1)						
Positive	10.5	52.6	21.1	5.3	89.5	.407 (.421)
No comment	5.3	0.0	0.0	0.0	5.3	
No response	0.0	5.3	0.0	0.0	5.3	
Response to the proposed electronic data recording/reporting format (Q3.2.4)						
Positive	10.5	15.8	21.1	5.3	52.6	.391 (.524)
Constructive feedback	5.3	15.8	0.0	0.0	21.1	
No comment	0.0	10.5	0.0	0.0	10.5	
No response	0.0	15.8	0.0	0.0	15.8	

*The p-value > 0.05 indicates there is no significant difference between midwives' responses with respect to factors affecting their ability to successfully complete the ANC tasks
